# Applying Metrics to Outpatient Oncology Advanced Practice Providers

**Published:** 2016-03-01

**Authors:** Elizabeth Gilbert, Victoria Sherry

**Affiliations:** Abramson Cancer Center, University of Pennsylvania, Philadelphia, Pennsylvania

**Applying Metrics to Outpatient Oncology Advanced Practice Providers**

A continuing education article for nurse practitioners, physician assistants, clinical nurse specialists, advanced degree nurses, oncology and hematology nurses, pharmacists, and physicians.

**Release date:** March 15, 2016

**Expiration date:** March 15, 2017

**Expected time to complete this activity as designed:** .5 hour

**Meniscus Educational Institute**

3131 Princeton Pike,

Building 1, Suite 205A

Lawrenceville, NJ 08648

Voice: 609-246-5000

Fax: 609-449-7969

E-mail: lrubin@meniscusedu.com

**Journal of the Advanced Practitioner in Oncology**

94 N. Woodhull Road

Huntington, NY 11743

Voice: 631-692-0800

Fax: 631-692-0805

E-mail: claudine@harborsidepress.com

© *2016, Meniscus Educational Institute. All rights reserved.*

## Faculty

**Elizabeth Gilbert, MS, PA-C,** Abramson Cancer Center, University of Pennsylvania, Philadelphia, Pennsylvania

**Victoria Sherry, MSN, CRNP, ANP-BC, AOCNP®,** Abramson Cancer Center, University of Pennsylvania, Philadelphia, Pennsylvania

## Activity Rationale and Purpose

Advanced practice providers (APPs) are assuming an increasing role in collaborative practice teams within oncology. Therefore, it is of utmost importance that they develop systems of measuring their contribution to the clinical practice and participation in patient care. Even though institutions and practices are using outcomes as benchmarks, many acknowledge they have not measured the impact of APP interventions. The demonstration of impact falls to the oncology APP by developing tools, guidelines, or methods to collect reliable metrics specific to their collaborative role in oncology.

Metrics will not only promote performance evaluation, improvement, and professional growth, but measuring productivity and quality will promote the value of APPs in the oncology setting, further highlight their value in the collaborative practice, and enhance their influence in quality care.

## Intended Audience

The activity’s target audience will consist of nurse practitioners, physician assistants, clinical nurse specialists, advanced degree nurses, oncology and hematology nurses, pharmacists, and physicians.

## Learning Objectives

After completing this educational activity, participants should be able to:

- Discuss metrics that could be monitored and benchmarked to highlight the contributions of the APP in his or her role as a member of the collaborative practice team in oncology

## Continuing Education

**Statement of Credit—Participants who successfully complete this activity (including the submission of the post-test and evaluation form) will receive a statement of credit.**

**Physicians.** The Meniscus Educational Institute is accredited by the Accreditation Council for Continuing Medical Education (ACCME) to provide continuing medical education for physicians.

The Meniscus Educational Institute designates this journal article for a maximum of 0.5 *AMA PRA Category 1 Credits*™. Physicians should claim only the credit commensurate with the extent of their participation in the activity.

**Nurses.** This activity for 0.5 contact hour is provided by the Meniscus Educational Institute.

The Meniscus Educational Institute is accredited as a provider of continuing nursing education by the American Nurses Credentialing Center’s Commission on Accreditation.

## Financial Disclosures

All individuals in positions to control the content of this program (eg, planners, faculty, content reviewers) are expected to disclose all financial relationships with commercial interests that may have a direct bearing on the subject matter of this continuing education activity. Meniscus Educational Institute has identified and resolved all conflicts of interest in accordance with the MEI policies and procedures. Participants have the responsibility to assess the impact (if any) of the disclosed information on the educational value of the activity.

**Faculty**

**Elizabeth Gilbert, MS, PA-C,** has nothing to disclose.

**Victoria Sherry, MSN, CRNP, ANP-BC, AOCNP®,** has nothing to disclose.

**Lead Nurse Planner**

**Wendy J. Smith, ACNP, AOCN®,** has nothing to disclose.

**Planners**

**Jeannine Coronna** has nothing to disclose.

**Claudine Kiffer** has nothing to disclose.

**Terry Logan, CHCP,** has nothing to disclose.

**Pamela Hallquist Viale, RN, MS, CNS, ANP,** has nothing to disclose.

**Lynn Rubin** has nothing to disclose.

**Content Reviewers**

**Glenn Bingle, MD, PhD, FACP,** has nothing to disclose.

**Karen Abbas, MS, RN, AOCN®,** has nothing to disclose.

**Wendy J. Smith, ACNP, AOCN®,** has nothing to disclose.

## Disclaimer

This activity has been designed to provide continuing education that is focused on specific objectives. In selecting educational activities, clinicians should pay special attention to the relevance of those objectives and the application to their particular needs. The intent of all Meniscus Educational Institute educational opportunities is to provide learning that will improve patient care. Clinicians are encouraged to reflect on this activity and its applicability to their own patient population.

The opinions expressed in this activity are those of the faculty and reviewers and do not represent an endorsement by Meniscus Educational Institute of any specific therapeutics or approaches to diagnosis or patient management.

## Product Disclosure

This educational activity may contain discussion of published as well as investigational uses of agents that are not approved by the US Food and Drug Administration. For additional information about approved uses, including approved indications, contraindications, and warnings, please refer to the prescribing information for each product.

## How to Earn Credit

To access the learning assessment and evaluation form online, visit www.meniscusce.com

**Statement of Credit:** Participants who successfully complete this activity (including scoring of a minimum of 70% on the learning assessment) and complete and submit the evaluation form with an E-mail address will be able to download a statement of credit.

## ARTICLE

Much of oncology care is now delivered through a team approach; understanding the potential benefits of the physician/advanced practice provider (APP) collaborative unit, in addition to the value of the APP individually, has never been more important. With the increased presence of APPs (nurse practitioners and physician assistants) in the delivery of health-care services, particularly in oncology, the importance of identifying and monitoring quality and productivity is key to the growth of these professionals to help maintain and encourage successful collaborations with physicians. One study demonstrated that 54% of oncologists work collaboratively with APPs (Erikson, Salsberg, Forte, Bruinooge, & Goldstein, 2007).

At the Abramson Cancer Center (ACC), a division of the University of Pennsylvania Health System (UPHS) and a National Cancer Institute (NCI)-designated comprehensive cancer center located in Philadelphia, 83% of the physicians collaborate with an APP. With the widening gap between the demand for oncology services and available providers, it is estimated that these numbers will continue to increase. Despite this clear upward trend, there are no benchmark metrics specific to the oncology APP that can be utilized to represent the value of these oncology professionals.

Quantifying, reporting, and comparing metrics are some of the tasks important to improving outcomes (Porter, 2010). Measuring productivity and quality through the use of metrics is a way for APPs to promote their worth and show their commitment to continuous quality improvement (Moote, Nelson, Veltkamp, & Campbell, 2012; Sollecito & Johnson, 2011). Advanced practitioners can create metrics that align with evidence-based practices to promote quality, improve patient safety, and reinforce best practices (Agency for Healthcare Research and Quality, 2013). An additional advantage to creating standards through the use of metrics is that the information gathered can improve professional work evaluations, provide guidelines for workload and compensation, and help recruit and retain quality employees.

Many areas of health care utilize evidence-based metrics to represent performance benchmarks; however, very little quality benchmarking exists for oncology APPs (Hinkel et al., 2010; Moote et al., 2012). The metrics being utilized in practice come from primary care settings and are not sufficienly tailored to be applicable to oncology (Moote et al., 2012). Examinations of specific oncology APP metrics have primarily been limited to patient satisfaction and productivity (as measured by the amount of patients seen, billings, and relative value units [RVUs] generated; Buswell, Ponte, & Shulman, 2009; Hinkel et al., 2010; Moote et al., 2012). Although these measures are a good start, they do not capture the varied role and professionalism of the APP, particularly in the outpatient oncology setting.

Like physicians, APPs are providers of care, so it is reasonable to define and track evidence-based APP-driven metrics in the way physicians do, by including quality indicators as well as the financial impact of care (Campion, Larson, Kadlubek, Earle, & Neuss, 2011; Makari-Judson, Wrenn, Mertens, Josephson, & Stewart, 2014). Advanced practitioners can then use this information to establish their contribution to their collaborative practices as well as provide feedback for learning, ongoing performance improvement, and professional growth.

## PROPOSED METRICS CARD

Part of the ACC’s mission is to enhance the patient experience through innovation and quality improvement (Terwiesch, Mehta, & Volpp, 2013). Research has shown that when the value of an individual can be assessed through a diverse set of metrics, a system of support for specific standards can be endorsed (Kennedy, Johnston, & Arnold, 2007). Gaining support for the standards APPs uphold is one of the goals of this project.

Although quality improvement is a major part of this institution’s mission, APPs have lacked a means to communicate the many ways they affect patient care and the health system. With more than 500 APPs in almost every medical subspecialty of the UPHS system and more than 30 specifically in the hematology/oncology division, a framework was needed to measure the quality care impact and professional growth of APPs.

Through the strong leadership of the Chief Administrative Officer of Cancer Service lines, Regina Cunningham, PhD, RN, AOCN®, a team of outpatient APPs formed a committee with the aim to search the literature for an applicable panel of APP-driven metrics to use within the hematology/oncology division. The team included APPs from medical oncology, hematology/oncology, internal medicine, and radiation oncology.

Determining which initial metrics to pilot was a complicated process. For the metrics to be meaningful, they needed to be diverse enough to encompass the many dimensions of the APP’s role across the various oncology specialties. To monitor and benchmark progress over time, it was essential that the metrics be easily trackable.

The APP committee chose metrics that represented four performance categories: financial impact, professional development, patient satisfaction, and quality indicators (specific to patient encounters; see Table). The selection of these metrics was made after a thorough review of the literature and developed using the evidence-based metric recommendations from a variety of professional oncology organizations: the American Society of Clinical Oncology (ASCO), the American Society for Radiation Oncologists (ASTRO), the National Comprehensive Care Network (NCCN), the National Quality Forum (NQF), the American Society of Hematology (ASH), and ASCO’s Quality Oncology Practice Initiative (QOPI).

**Table:**
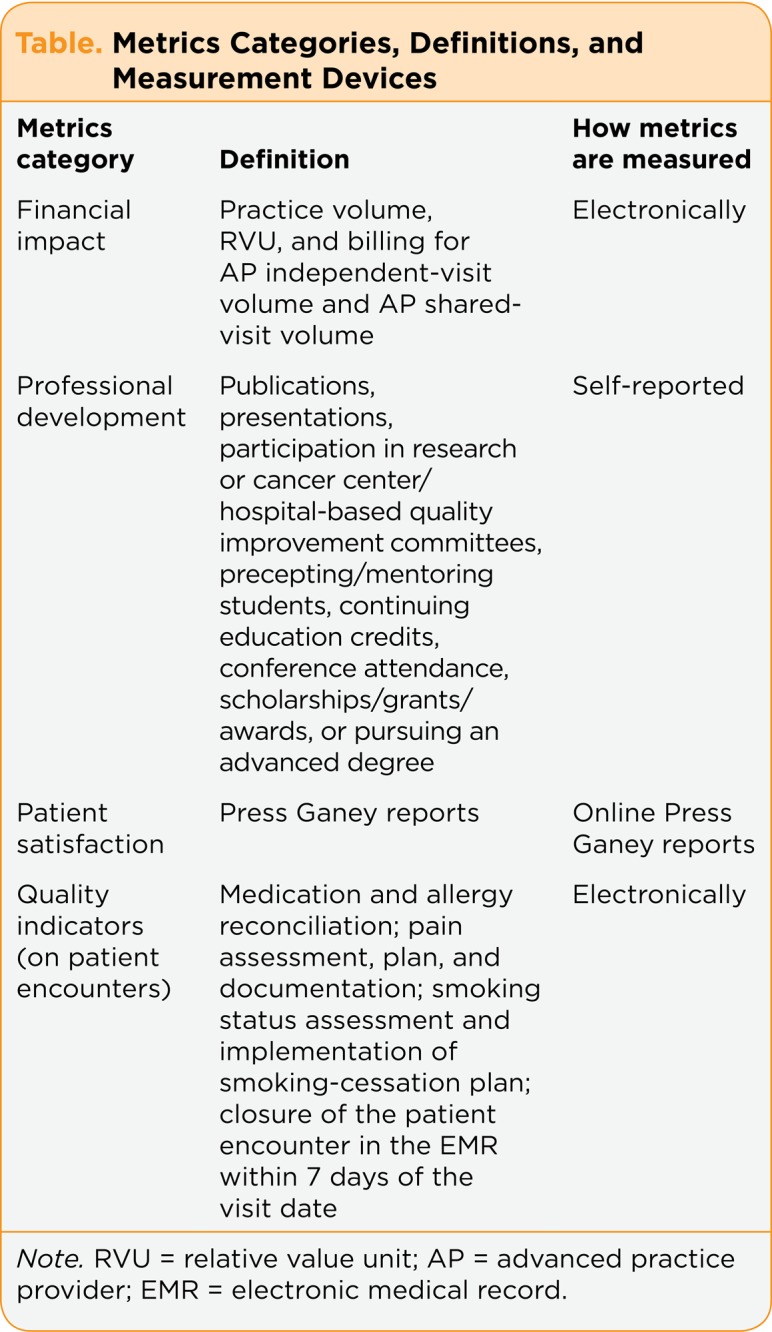
Metrics Categories, Definitions, and Measurement Devices

## EXPLANATION OF INDIVIDUAL METRICS

**Financial Impact**

Understanding and benchmarking financial productivity are essential in any profession. High or low values in this category can help to illuminate the areas of practice that are working well and those that may need revision. Metrics in this category can also help establish workload standards and be a stepping stone to developing incentive programs related to performance that are similar to those for physicians (Cassel & Jain, 2012). Included in this category are total practice volume, number of independent and shared patient encounters by the APP, relative value units for independent APP patient encounters, and billings generated by the APP and the practices they support.

Importance of Shared-Visit Reporting: Collaborative styles have been examined and documented in multiple articles (Towle et al., 2011; Buswell et al., 2009). For the purposes of this article, the terminology from Buswell et al. (2009) will be used to describe models of care delivery: independent-visit model (IVM), shared-visit model (SVM), and mixed-visit model (MVM).

Understanding that there are different models of care delivery used by APPs, and that billed services performed by APPs are not always billed in their name, it is apparent that using standard measures of productivity such as independent encounter volume and billing undervalues the APP contribution. Accurate measurement within a financial impact category relies on a system that not only credits the work billed independently by the APP, but also recognizes some of the significant work bundled and billed under the physician’s name.

**Figure 1:**
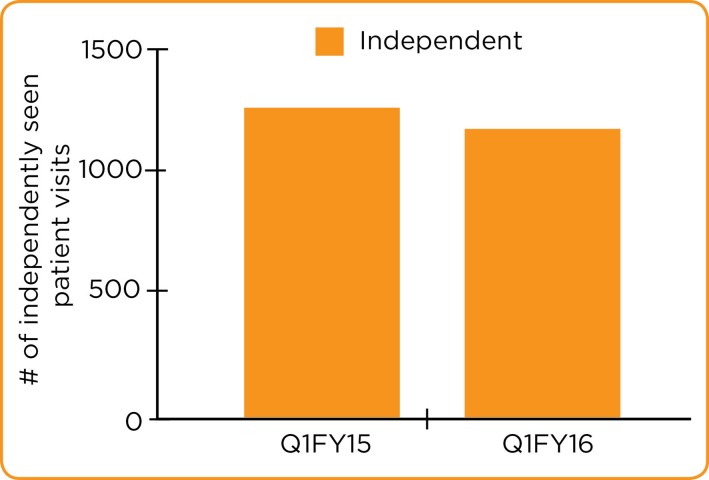
Measuring APP productivity using only independent-visit data. Q1FY15 = before measuring metrics; Q1FY16 = after defining, educating, and measuring metrics.

The ACC only captured the financial impact from independent billings and patient encounters by the APP, yet many of the collaborative practices functioned in the SVM or MVM. Utilizing these models often led to billing under the physician’s name. By including "shared-visit" data, APP patient visits can be monitored more completely, and the overall contributions to practice productivity can be more transparent to cancer center leadership, collaborating physicians, and colleagues. Therefore, shared-visit data are an invaluable addition to the APP financial category; without them, much of the APP’s work is otherwise unaccounted for (see Figures 1 through Figure 3).

**Figure 2:**
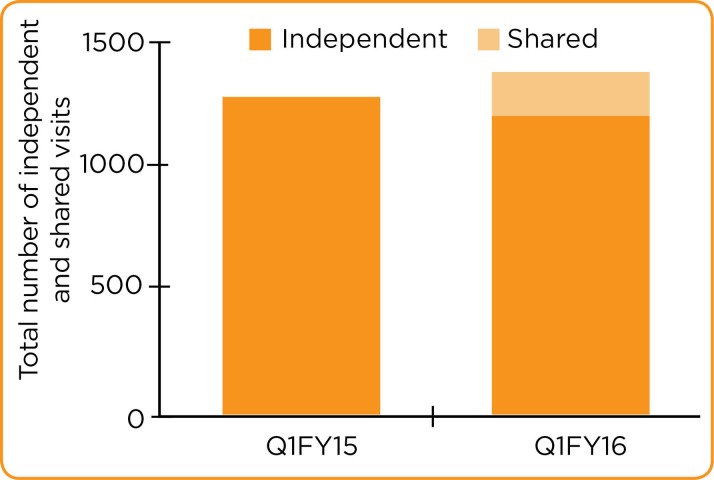
Using metrics to identify APP work not designated as an independent visit. Q1FY15 = before measuring metrics; Q1FY16 = after defining, educating, and measuring metrics.

  

**Figure 3:**
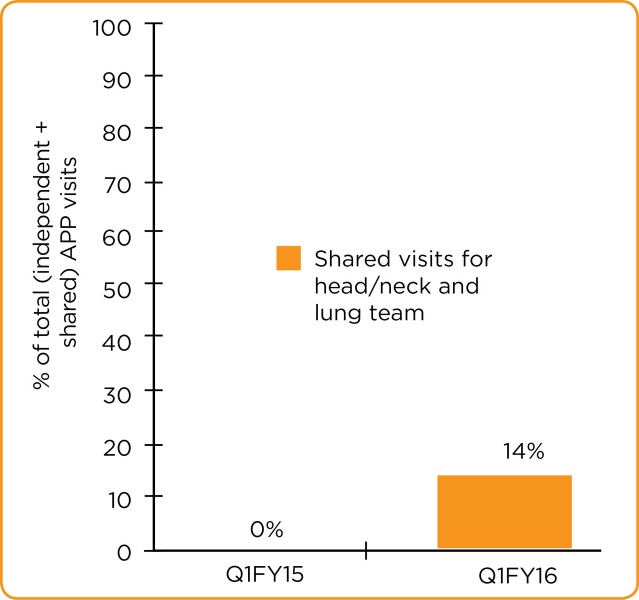
Percentage of shared visits identified in APP workload. Q1FY15 = before measuring metrics; Q1FY16 = after defining, educating, and measuring metrics.

Data from Figures Figure 1, Figure 2, and Figure 3 demonstrate the importance of measuring more than just independent-visit data for our head/neck/lung specialty APPs. If shared visits were not captured, APP productivity appears to drop (Figure 1). However, as shown in Figures 2 and 3, APP productivity actually increased because there was a shift in how the patients’ visits were accomplished, not that the APPs were "less" productive.

The APP metrics committee formulated the definitions of a shared visit. It was a difficult task, but it was clear that shared work could be defined by a few common factors. The committee determined that for a patient encounter to be deemed a shared visit, the APP must physically interact with the patient during the encounter as well as perform any number of elements of the encounter (i.e., obtaining the patient’s history; formulating/documenting the plan; ordering and following up on medications, labs, procedures, radiology, and scan reports; care coordination; and/or teaching).

**Professional Development**

Clinical knowledge and skills are important components in the certification and advancement of the APP (Hooker, Carter, & Cawley, 2004). As APPs are lifelong learners, professional development is their responsibility to become proficient, expert practitioners (Jasper, 2011). Professional development encourages APPs to seek out new information and build on existing knowledge.

At UPHS, in addition to the mandatory hours of continuing education credits, professional development was measured through documentation of the following items: publications, presentations, participation in research activities, precepting/mentoring students, conference attendance, scholarships/awards, pursuing an advanced degree, and/or serving on quality-improvement committees.

**Patient Satisfaction**

With health care’s emphasis on patient-centered care, measuring patient satisfaction is crucial to define patient perceptions of health-care quality (Sofaer & Firminger, 2005). Feedback regarding patients’ visit experiences helps to address their needs effectively. Patient surveys, such as Press Ganey, are used to assist in understanding how satisfied the patient populations are in all facets of care (Chandra et al., 2011). Press Ganey’s stated mission is to "support health care providers in understanding and improving the entire patient experience" (Press Ganey, 2015). The opinions expressed by patients receiving care give the APPs an opportunity to see their strengths and areas where the quality of care needs to be improved.

**Quality Metrics on Patient Encounters**

Quality indicators can be defined as measures of health-care quality and patient safety (Boulkedid et al., 2011). They provide systematic measurement, monitoring, and reporting necessary to make salient advances in improving care.

The quality indicators chosen included process metrics for both independent and shared patient visits. The four key metrics selected included documentation and reconciliation of medication and allergy lists; pain assessment, plan, and documentation; smoking status assessment and implementation of smoking cessation plan; and closure of the patient encounter in the electronic medical record (EMR) within 7 days of the visit date.

Medication reconciliation and allergy documentation were included as metrics because when performed, they are associated with a dramatic reduction in medication errors, prevention of potential adverse drug events, and thus increased patient safety and decreased health-care costs (Barnsteiner, 2008; Aspden, Wolcott, Bootman, & Cronenwett, 2007). Accurate medication reconciliation also helps the provider monitor patient adherence and therapeutic response as well as allows for continuity of care across different disciplines in the health-care system.

Medication reconciliation is especially critical with oncology patients. Medications and cancer treatments must be accurately documented and relayed to other health-care providers due to the unique side effects and potential drug interactions with any cancer therapy the patient is receiving.

Evaluation of pain was included because it occurs in approximately 70% to 80% of patients and is one of the most frequent and disturbing symptoms (Caraceni et al., 2012). There is increasing evidence that adequate pain management is directly linked to improvement in quality of life (Temel et al., 2010). Effective evaluation and treatment of cancer pain can ameliorate unnecessary suffering and provide support to the patient and family. Pain management is an essential part of oncologic care to maximize patient outcomes (NCCN, 2015).

Smoking is the leading preventable cause of death in the United States (American Lung Association, 2014). Smoking is linked to a variety of cancers, including lung, head & neck, bladder, esophageal, stomach, uterine, cervical, colon, rectal, ovarian, and acute myeloid leukemia (American Cancer Society, 2015). Continued smoking after having been diagnosed with cancer has many negative consequences, such as reduced effectiveness of treatment, decreased survival time, and risk of recurrence (de Bruin-Visser, Ackerstaff, Rehorst, Retel, & Hilgers, 2012; Piper, Kenford, Fiore, & Baker, 2012). Smoking cessation is associated with improved prognostic outcomes, increased quality of life, and decreased health-care costs (Villanti, Jiang, Abrams, & Pyenson, 2013). Smoking cessation assessment and counseling are important elements in cancer care, and ones that APPs can drive.

The quality of health care across the continuum depends on the integrity, dependability, and succinctness of health information. Prompt completion and closure of all outpatient encounters are mandatory for clinical, quality, legal, and billing compliance reasons (University of Pennsylvania Health System, 2007). Providers may not submit a claim to Medicare until the documentation for a service is completed (Centers for Medicare & Medicaid Services [CMS], 2015; Pelaia, 2007). The CMS (2015) expects documentation from practitioners to occur "during or as soon as practical after it is provided in order to maintain an accurate medical record." The UPHS determined that requiring completion of documentation in the EMR within 7 days would fulfill CMS recommendations. Chart closure is not only important from a financial perspective, but it also optimizes patient care and improves outcomes (CMS, 2015).

## OUTCOMES AND NEXT STEPS

The initial pilot of the metric report was performed in the head/neck and lung group to prove the feasibility of collecting metric data. Shared-visit data was recorded manually and cross-checked with the electronic report. Teaching and reeducation on completing quality metrics were reviewed with each APP. Accurate reports were generated, and the process was disseminated to the entire hematology/oncology outpatient division. Benchmarking is currently in progress and is continually being refined from colleague feedback.

**Figure 4:**
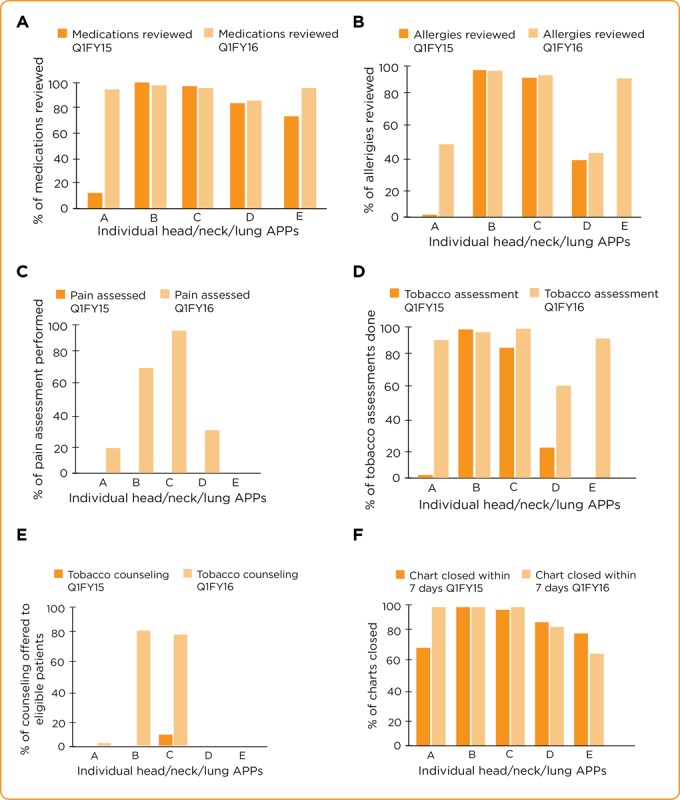
(A) Medication review results for all shared and independent visits. (B) Allergy review results for all shared and independent visits. (C) Pain assessment results for all shared and independent visits. (D) Tobacco assessment results for all shared and independent visits. (E) Tobacco counseling results for all shared and independent visits. (F) Chart closure results for all independent visits only. Q1FY15 = before measuring metrics; Q1FY16 = after defining, educating, and measuring metrics.

The next step is to set an initial benchmark for each metric proposed (i.e., ensuring that all APPs achieve an 80% or higher on the quality metrics) and work with the APPs to use the information to improve practice issues within the division. Figures 4A through Figures 4F show the results of the initial monitoring. Most of the metrics show dramatic improvements with individual APPs, whereas others recorded similar or slightly decreased results. Certain results clearly show that there are problems with the usability of the metric or that there is an APP knowledge deficit regarding proper utilization. Creating a system for auditing the metric results will ensure ongoing quality control and identify areas that need reinforcement.

## CONCLUSION

**Figure 5:**
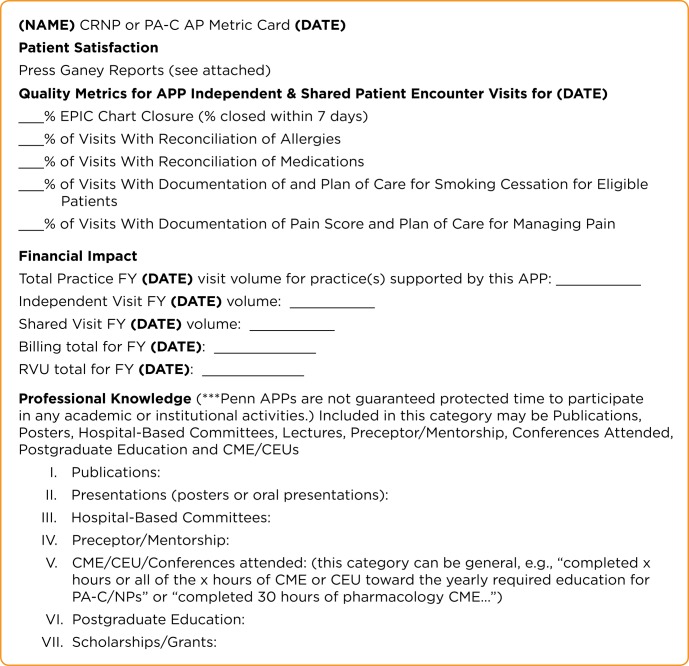
Abramson Cancer Center Individual APP Metrics Card: Initial Version.

It is important to measure and show the quality of care and productivity within collaborative oncology practices. Creating evidence-based metrics in a diverse set of categories better illuminates the significance of APP contributions. Prior to establishing these metrics, each APP within the group received one generic yearly evaluation, with subjective feedback from his/her collaborating physician(s) and supervisor. Figure 5 illustrates a sample template metric card for each APP. These metrics now provide the tangible framework necessary to demonstrate the contributions of advanced practice providers, enable a standard to ensure the quality of care for all patients, and encourage professional growth.
